# Using refractive lenses to provide a variable spot size for Kirkpatrick–Baez mirrors

**DOI:** 10.1107/S1600577518010603

**Published:** 2018-08-21

**Authors:** Steve M. Heald, Eric M. Dufresne

**Affiliations:** a XSD-APS, Argonne National Laboratory, Building 435E, 9700 South Cass Avenue, Argonne, IL 60439, USA

**Keywords:** X-ray microprobe, Kirkpatrick–Baez mirror, refractive lenses

## Abstract

For many X-ray microprobe experiments it is desirable to be able to vary the beam size: using large beams for survey scans and a small beam for the final measurements. Beryllium refractive lenses were found to be a simple and controllable method for enlarging the focus in a Kirkpatrick–Baez-based microprobe. They can provide variable spot size, can be quickly inserted or removed and do not move the beam center on the sample.

## Abstract 2

Refractive lenses are used to vary the focal spot size for a Kirkpatrick–Baez X-ray microprobe.

## 1. Introduction   

**Figure 1:**
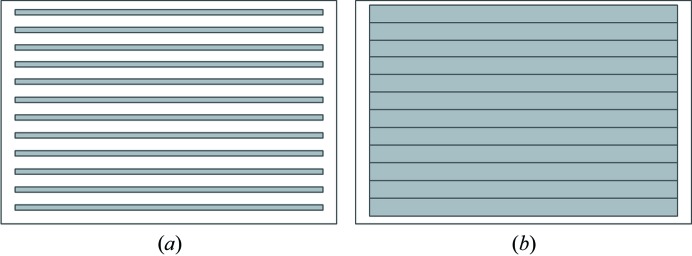
Illustrating two possible methods for scanning of large areas with a microbeam: (*a*) the undesirable case using large scan intervals with a small beam and (*b*) the better solution using an expanded beam to avoid missing parts of the sample.

Kirkpatrick–Baez (KB) mirror systems (Eng *et al.*, 1998 ▸; Kirkpatrick & Baez, 1948 ▸) are widely used in microspectroscopy owing to their excellent focusing and achromatic properties. A common application is the study of environmental or geoscience samples that are highly inhomogeneous. In these cases it is often necessary to scan large areas to find interesting regions for spectroscopy. Even with continuous or quick scanning and short integration times, it can be time consuming to scan large areas with micrometre-sized beams. Thus, the experimenter may end up using scan steps that are larger than the beam size, leading to the situation illustrated in Fig. 1 ▸(*a*). Small structures may be missed using such a scan. A more desirable situation would be to increase the beam size to allow full coverage in a reasonable time as shown in Fig. 1 ▸(*b*). There are several ways to vary the focused beam size. The mirror bending or angle can be varied to degrade the focus, which can result in movement of the beam such that, after returning to the small spot, the position is no longer registered with the large-spot image. Another approach would be to move the sample upstream or downstream of the focus where the beam is larger. However, for confocal or wavelength-dispersive detectors this would result in the sample being outside of the detector focus. Some beamlines can control the focus size by positioning the KB-mirror source at an intermediate focal spot (Heald, 2002 ▸; Marcus *et al.*, 2004 ▸), the size of which can be varied by a slit. This is a nice solution that can work well, but for a highly brilliant source can be inefficient. For example, for the new multi-bend achromat rings currently coming into operation, the brilliance is such that the entire output can be focused to a few hundred nanometres. Thus, using a slit to vary the spot size would mean reduced flux for the small beam sizes. It is also possible to manufacture a mirror with multiple lanes, each providing a different focal spot size (Laundy *et al.*, 2016 ▸). In this work we demonstrate that refractive X-ray lenses (Snigirev *et al.*, 1996 ▸; Lengeler *et al.*, 2002 ▸) can be combined with typical KB mirrors to provide a convenient and controllable spot-size expansion. Since they are on-axis optics, they can be rapidly inserted or removed without moving the center of the focus.

**Figure 2:**
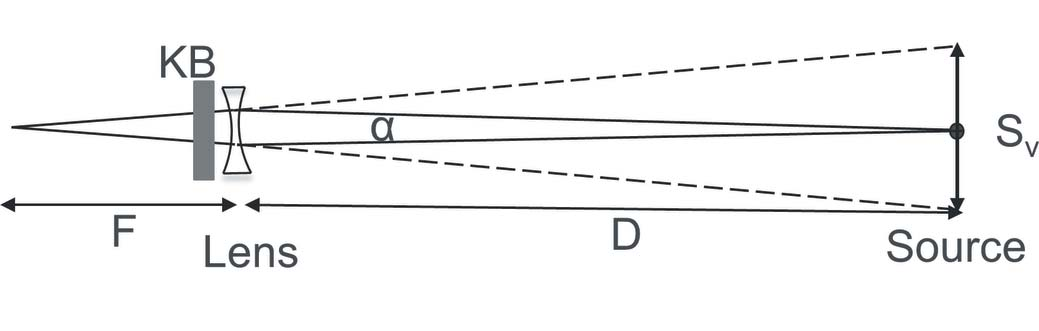
The geometry for estimating the virtual-source size *S*_v_ seen by the KB mirrors. The lens was placed close to the KB mirrors indicated by the gray box. *D* is the distance from the source point being imaged by the KB mirror to the lens and *F* is the focal length of the lens. α is the acceptance angle of the lens, typically defined by the entrance slits to the KB mirrors. In this work, *D* = 56.5 m, *F* varies from 4.4 m to 9.4 m (see Table 1 ▸), and the KB mirrors are placed close to the lens at 57 m from the virtual source.

The basic idea is illustrated in Fig. 2 ▸. A slightly focusing refractive lens is placed in front of the KB mirrors; this converts the diverging beam into a slightly converging beam that can be traced back to the source point being imaged by the mirrors. This new source is greatly enlarged resulting in a much larger KB mirror focus. If the focal length of the lens is *F*, the distance to the source being imaged by the KB mirror is *D* and the acceptance angle of the KB mirror is α, then the virtual source seen by the mirrors has a size given by
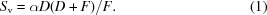
The mirror focus is correspondingly enlarged. The refractive lenses are chromatic with a focal length varying as the square of the energy and the spot size will have an energy dependence, but in most applications the spectroscopy would be performed with the lens removed using a small spot.

## 2. Experiment   

**Figure 3:**
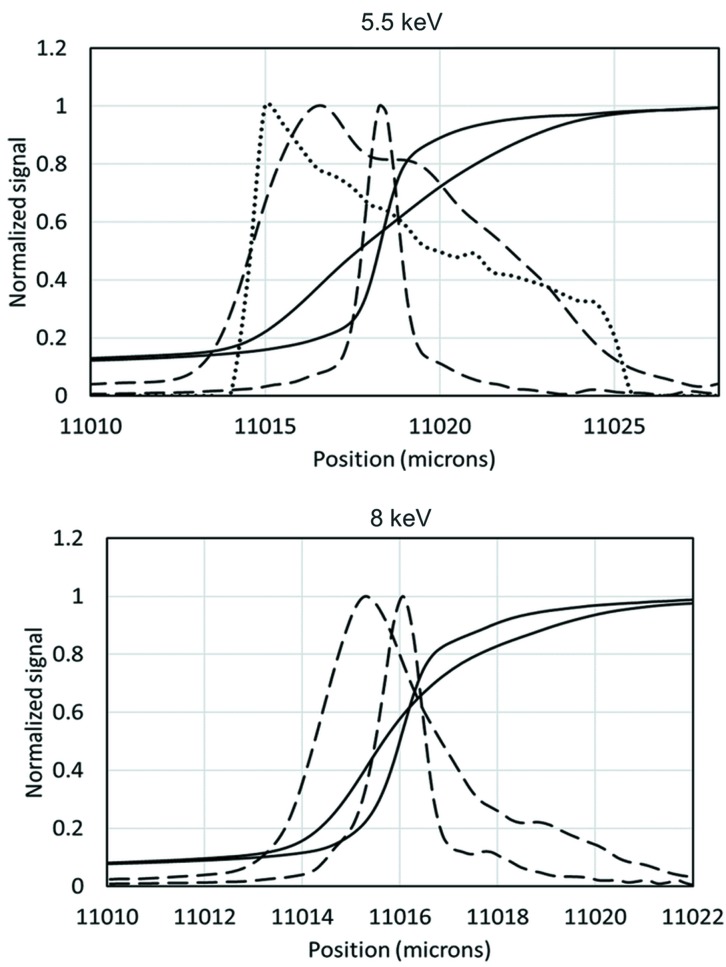
The solid lines are wire scans in the vertical, measuring the focal spot size with and without the lens inserted. Dashed lines are derivatives of the solid curves. The transmitted signals and derivatives are normalized to a value of 1 for comparison. For the 5.5 keV case, the dotted line shows the ray-tracing result.

To test the idea we inserted two one-dimensional parabolic Be lenses (RXOPTICS, Juelich, Germany) with radii of 0.2 mm in front of the KB-mirror based microprobe at beamline 20-ID at the Advanced Photon Source (Heald *et al.*, 1999 ▸). The KB mirrors were 57 m from the source with an entrance slit of 180 µm, giving an acceptance angle of α = 3.15 µrad. The cylindrical lenses were oriented to expand the vertical beam source size, and were placed 0.5 m from the KB mirror at 56.5 m from the virtual source position. Expansion of only the vertical is sufficient, since the beamline sample stage employs continuous scanning in the horizontal, and the horizontal resolution can be controlled by the scan-speed and readout intervals. The beam size was measured by scanning a 100 µm W wire through the beam while recording the transmitted signal with an ion chamber. The results are shown in Fig. 3 ▸ for two different beam energies. The unperturbed beam size of slightly more than 1 µm was expanded to several micrometres with the lenses. In both cases the knife-edge scans cross near to their midpoint, showing that the beam position is almost unchanged with the insertion of the lens. The attenuation of the beam by the lenses was small. For the worst case at 5.5 keV, the transmitted beam retained 91% of the incident intensity.

The expanded size varied with energy since the focusing power of the lenses varied. The expected focus size can be estimated using the idea demonstrated in Fig. 2 ▸ with equation (1) and the demagnification factor of the mirror, which was 270. These results are shown in Table 1 ▸. The angle and position of the rays from the virtual source will be highly correlated. Thus, it cannot be treated as a simple Gaussian-shaped source when determining its size. Therefore, in the table we used two different measures of the focal spot size: the full width at half-maximum (FWHM) of the knife-edge derivative and the 10–90% transmission values in the knife-edge scan. The 10–90% measurement is used as an estimate of the entire beam size. The results show that the simple picture in Fig. 2 ▸ gives a reasonable estimate of the total width of the beam, and the 10–90% measure is the best comparison. The agreement would probably be even better if the estimated spot calculation included other sources of broadening such as the figure errors in the mirrors. These would have a larger effect on the smaller spot sizes.

The expanded beams are somewhat asymmetric, as expected from the geometry. The lens shifts the intersection points of the extreme rays on the mirror. There is a similar shift at each end of the mirror, but the curvature of the highly demagnifying elliptical mirror is much stronger on its downstream end. This gives a larger deflection for the rays hitting the downstream end of the mirror and an asymmetric beam. Ray-tracing using *Shadow* (Rebuffi & Sanchez del Río, 2016 ▸) verifies this simple picture as shown in Fig. 3 ▸ for the 5.5 keV case. This calculation assumes an ideal mirror and will be additionally spread out by the slope errors of a real mirror. We did not try to include the slope errors in this case since they were not measured, but the ray-tracing shows a similar asymmetric shape to the measurements. An asymmetric spot is not a problem since the main goal is to spread the beam in order to avoid missing small features.

## 3. Conclusions   

We have shown that insertion of refractive lenses is a convenient method for expanding the spot of a KB focused beam. They do not shift the center of the beam, can be rapidly inserted or removed and the final beam size can be estimated with a simple calculation. Their primary disadvantage is a beam size that depends on energy, but in many cases a survey scan with the larger beam is followed by detailed spectroscopic studies with the original achromatic small spot.

## Figures and Tables

**Table 1:** Comparison of the measured spot size with the estimated spot size, using equation (1) to calculate the expansion of the source size as illustrated in Fig. 2 ▸. Focal lengths for the lens were calculated using the online calculator from RXOPTICS (http://www.rxoptics.de/parameters.html)

Energy (keV)	Derivative FWHM (µm)	10–90% beam width (µm)	Lens focal length (m)	*S*_v_, effective source size (µm)	Estimated focal spot (µm)
5.5	7.5	8.4	4.4	2460	9.1
6.5	5.5	6.8	6.2	1640	6.1
8.0	2.6	5.3	9.4	1250	4.6
